# What is the association of heme aggregates with the peritrophic matrix of adult female mosquitoes?

**DOI:** 10.1186/1756-3305-7-362

**Published:** 2014-08-13

**Authors:** Tereza Magalhaes

**Affiliations:** Department of Virology and Experimental Therapeutics, Centro de Pesquisas Aggeu Magalhães, Fundação Oswaldo Cruz, Av. Moraes Rego s/n - Cidade Universitária, CEP 50740-465, Recife, PE Brazil

**Keywords:** Peritrophic matrix micrographs, Iron aggregates, Detoxifying layer, Mosquitoes

## Abstract

The aim of this Letter is to call attention to the presence of iron-containing or heme aggregates seen within or near the peritrophic matrix (PM) in published *Anopheles gambiae* and *Aedes aegypti* midgut micrographs. The micrographs shown here suggest that the PM of *An. gambiae* adult female is not a homogenous layer and instead may have a peritrophin layer surrounded by heme aggregates that are possibly associated to other molecules involved in their formation. In depth studies addressing PM structure in different mosquito species are needed, as these will be important to continue clarifying the roles of the PM.

When adult female mosquitoes take a blood meal for egg production, an extracellular layer produced by midgut epithelial cells is formed around ingested blood, where it remains in the same location until it is degraded. This layer is called type 1 peritrophic matrix (PM) and its production is stimulated by distension of the midgut that naturally occurs after a blood meal [1]. The PM is composed of chitin fibrils, proteins and proteoglycans [1], which interact in an apparently sophisticated but unclear manner to form a 3-dimensional structure. The PM of *Aedes aegypti* and *Anopheles gambiae* mosquitoes differs in a few aspects such as formation kinetics, and the number and mode of production of proteins. While the *An. gambiae* PM appears to have approximately 40 major proteins, fewer PM proteins have been identified in *Ae. aegypti* (around 20) [2]. An in depth proteomic analysis of the *An. gambiae* PM broadened the number of PM proteins in this species [3], and it is possible the same could occur with *Ae. aegypti*. As for protein production, *An. gambiae* PM proteins are stored in vesicles of epithelial cells and are promptly secreted after a blood meal, while in *Ae. aegypti* PM proteins need to be synthesized after blood ingestion [2]. Following an increase in the number of studies on insect PMs, different types of molecular structure models have been proposed [3, 4]. These models are based on the characterization of PM proteins and their putative interaction with molecules such as chitin fibrils and digestive enzymes secreted by the midgut epithelium. They also consider the putative functions of insect PMs, such as controlling the traffic of molecules between midgut epithelium and lumen, and protection of the midgut epithelium against mechanical abrasion and toxic products derived from blood digestion (e.g. heme). Whilst several putative roles have been ascribed to the PM of different mosquito species, various aspects of the molecular structure and functions of mosquito PM remain undefined. For instance, a study using *Ae. aegypti* shows no effect on mosquito survival, fecundity or protection against pathogens when PM formation is hindered [5]. Results on the protective role of mosquito PM against pathogens have also varied among studies.

The mosquito PM is a physical structure and can be physically isolated. In regards to PM micrographs, several studies show the mosquito midgut epithelium and PM under different conditions/treatments and magnification. As there is no described standard staining for insect PMs, authors have used different staining methodologies and visualized it as a sheet between the midgut epithelium and blood bolus. The appearance and description of the PM varies among studies, even within the same mosquito species, as different markers interact with different PM molecules. Pascoa *et al.* [6] demonstrated the possible role of *Ae. aegypti* PM to serve as a detoxifying barrier. In this study, the authors state that in an unstained micrograph (240× magnification) the PM looks darker than the midgut epithelium and ingested blood because of heme bound to it. Heme is derived from hemoglobin digestion. When *Ae. aegypti* midgut is stained with 3,3′-diaminobenzidine (DAB) for detection of heme peroxidase activity, the reaction produces a dark brown precipitate seen by light microscopy, which confirms the authors’ assumption that heme is bound to the PM. In this study, the PM is referred to whenever heme peroxidase activity is observed. Micrographs (640× magnification) of *Ae. aegypti* PM at several time-points (11, 22, 30, and 48 h) post blood meal (pbm) show a darker sheet between the midgut epithelium and ingested blood in the lumen. At 11 h pbm the PM looks thin and two dark layers can be seen. At 48 h, corresponding to a time-point where blood digestion is in an advanced stage, the dark staining looks amorphous throughout the midgut lumen, as if the PM is already degraded. Another study shows the association of an *Ae. aegypti* mucin-like molecule (AeIMUC1) to the PM. In electron micrographs of midguts dissected at 12 and 24 h pbm the PM is seen between the midgut epithelium and midgut lumen with heme aggregates observed “below” it [7]. Thus, according to the authors, these aggregates are not associated to the PM, which may sound contradictory to the PM-associated heme peroxidase activity in *Ae. aegypti* demonstrated by Pascoa *et al.* [6].

As for *An. gambiae* PM, Devenport *et al.* [8] shows the PM as a thin and undefined layer in midguts dissected at 12 and 24 h pbm (electron micrographs). In these micrographs, authors call attention to dark aggregates seen in the lumen near the PM and attribute these aggregates to “iron-containing byproducts of digestion”. In this case, the authors also do not associate the aggregates to the PM. The same description is found for aggregates observed near *An. gambiae* PM in Devenport *et al.* [9], however, one could claim that the aggregates can be seen within the PM in some of the micrographs.

Here, two types of *An. gambiae* micrographs are shown. In the first (Figure 1, 200× magnification), tissues were embedded in paraffin and incubated with mouse polyclonal anti-AgAper1 sera and then with anti-mouse IgG antibody conjugated with FITC as secondary antibody. In these samples, a thin green fluorescent layer corresponding to *An. gambiae* peritrophin 1 staining can be observed, similar to the micrographs published by Devenport *et al.* [8] showing stained AgAper1 within the mosquito PM.

**Figure 1:**
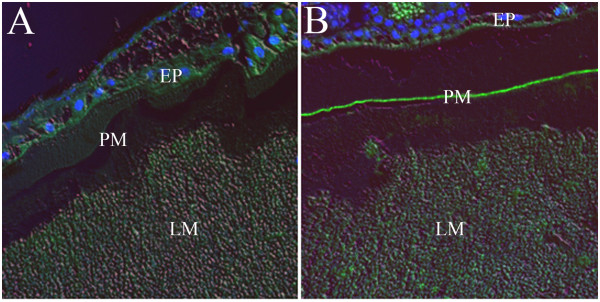
**Immunofluorescence of** ***Anopheles gambiae*** **abdomen at 24 h post-blood meal.** Paraffin-embedded sections were incubated with sera from naïve mice **(A)** or mice immunized with AgAper1 **(B)** as primary antibody, and with anti-mouse IgG-FITC as secondary antibody. Arrow in B shows AgAper1 fluorescence within the peritrophic matrix. Mounting media containing DAPI was used for nuclear staining of epithelial cells. Images were taken with a monochromatic camera using DIC, 480 nm, and 546 nm light. They were then false-coloured and merged to produce a single image. EP: epithelium; PM: peritrophic matrix; LM: midgut lumen containing undigested red blood cells. Magnification: 200×.

The second type of micrograph (Figure 2) shows *An. gambiae* midguts dissected at 24 h and 48 h post-blood meal, embedded in Araldite and stained with toluidine blue (1000× magnification). In these, the layer between the midgut epithelium and midgut lumen, which probably corresponds to the PM, looks much thicker than the AgAper1 immunofluorescence and contains what appear to be heme aggregates.

**Figure 2:**
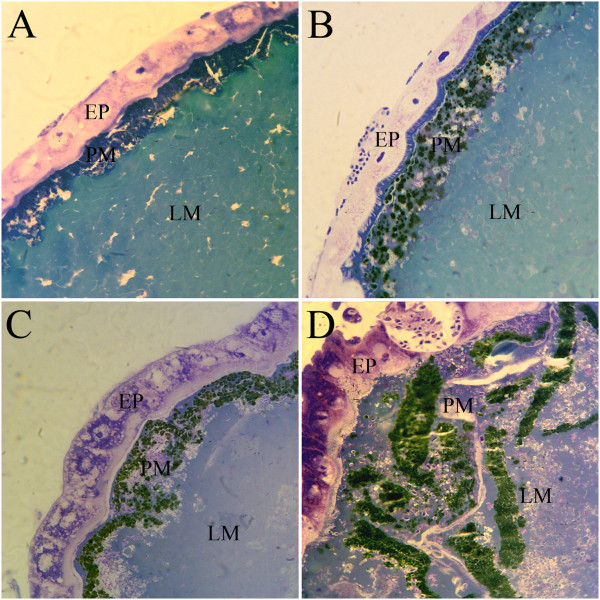
**Light micrographs of** ***Anopheles gambiae*** **midguts dissected at 24 h (A and C) or at 48 h post-blood meal (B and D).** Sections were immersed in Araldite and stained with toluidine blue after sectioning. EP: epithelium; PM: peritrophic matrix; LM: midgut lumen. Magnification: 1000×.

Taken together, the micrographs shown here suggest that: the localization and distribution of *An. gambiae* peritrophin 1, and possibly of other peritrophins, does not reflect the full extent of the PM, which is reasonable to assume; and that the PM is not an homogenous layer, but may have a peritrophin layer surrounded by heme aggregates that are possibly associated to other molecules involved in the formation of these aggregates.

This letter does not intend to prove that heme is associated to *An. gambiae* PM, but to raise questions in regards to this issue considering the presence of iron-containing (as some authors name it) or heme aggregates seen in various *An. gambiae* and *Ae. aegypti* midgut micrographs. In depth studies addressing PM structure in both mosquito species are needed, including experiments on chitin versus peritrophin staining, as these will be important to continue clarifying the roles of the PM in these insects.
